# The Spatial Precision of Contextual Feedback Signals in Human V1

**DOI:** 10.3390/biology12071022

**Published:** 2023-07-20

**Authors:** Lucy S. Petro, Fraser W. Smith, Clement Abbatecola, Lars Muckli

**Affiliations:** 1 Centre for Cognitive Neuroimaging, School of Psychology and Neuroscience, College of Medical, Veterinary and Life Sciences, University of Glasgow, Glasgow G12 8QB, UK; clement.abbatecola@glasgow.ac.uk; 2 Imaging Centre for Excellence (ICE), College of Medical, Veterinary and Life Sciences, University of Glasgow, Glasgow G51 4LB, UK; 3 School of Psychology, University of East Anglia, Norwich Research Park, Norwich NR4 7TJ, UK; fraser.smith@uea.ac.uk

**Keywords:** functional magnetic resonance imaging, cortical feedback processing, visual occlusion, top-down projection, predictive field

## Abstract

**Simple Summary:**

Higher levels of our visual systems process information from broad regions of our visual space, recognising scenes, objects, and faces, often regardless of their location. In contrast, earlier levels of our visual system respond to small detailed visual features, such as lines oriented in a certain direction and at precise spatial locations. High- and low-level visual areas are reciprocally connected, and visual perception emerges from recurrent neuronal computations between these areas. When tuning our brain imaging analysis to the spatial precision of the top-down connections from higher- to lower-level visual areas, do higher areas project their coarse spatial tuning or is this processing translated to the high-precision coding of the lower-level target region they are projecting to? For example, while neurons in early visual areas respond to the precise location of a cup on your desk, and higher-level areas continue to respond to the cup even when the cup is slightly moved, how is communication between higher-level visual areas and lower-level visual areas resolved? We hypothesised that the more generalised contextual information of ‘the cup is somewhere here’ is broadcast to cells in lower visual areas that would otherwise not respond, and those that are responding with the information ‘an object boundary is precisely detected here’ can now confirm whether it is consistent with the explanation that this contour belongs to a cup. In line with this hypothesis, we found that early visual areas receive feedback information that generalises across images in which the features have been spatially displaced. The data inform our understanding of how early visual areas are modulated by higher areas during visual perception.

**Abstract:**

Neurons in the primary visual cortex (V1) receive sensory inputs that describe small, local regions of the visual scene and cortical feedback inputs from higher visual areas processing the global scene context. Investigating the spatial precision of this visual contextual modulation will contribute to our understanding of the functional role of cortical feedback inputs in perceptual computations. We used human functional magnetic resonance imaging (fMRI) to test the spatial precision of contextual feedback inputs to V1 during natural scene processing. We measured brain activity patterns in the stimulated regions of V1 and in regions that we blocked from direct feedforward input, receiving information only from non-feedforward (i.e., feedback and lateral) inputs. We measured the spatial precision of contextual feedback signals by generalising brain activity patterns across parametrically spatially displaced versions of identical images using an MVPA cross-classification approach. We found that fMRI activity patterns in cortical feedback signals predicted our scene-specific features in V1 with a precision of approximately 4 degrees. The stimulated regions of V1 carried more precise scene information than non-stimulated regions; however, these regions also contained information patterns that generalised up to 4 degrees. This result shows that contextual signals relating to the global scene are similarly fed back to V1 when feedforward inputs are either present or absent. Our results are in line with contextual feedback signals from extrastriate areas to V1, describing global scene information and contributing to perceptual computations such as the hierarchical representation of feature boundaries within natural scenes.

## 1. Introduction

Neuronal receptive field properties in the primary visual area (V1) of rodents, cats, and monkeys have been investigated using invasive electrophysiological measures, e.g., [1,2,3]. In the human visual cortex, receptive field sizes have been assessed using subdural surface electrodes [4], population receptive field measures during functional magnetic resonance imaging (fMRI) [5], cortical-depth-dependent investigations during sub-millimetre fMRI [6], and fMRI in combination with computational modelling [7]. These approaches tend to yield consistent estimates in V1 of small (1–2 degrees), locally tuned, stimulus-dependent receptive fields. However, response properties of V1 neurons are also determined by cortical feedback inputs, such as from nearby areas V2–V4, the spatial extent of which is unknown in humans. Given the gradient of increasing receptive field sizes in ascending levels of the visual system (also across species e.g., [8]), we can speculate that cortical feedback signals might have the same spatial resolution as the receptive fields from their source higher-level visual area that generates the top-down feedback signal. Thus, the spatial resolution of a given feedback signal might be a proxy for its origin. Such a finding would inform computational and empirical frameworks describing how sensory neuronal information processing is contextualised or predicted by higher cortical areas.

To test the contribution of cortical feedback processing to feedforward processing, it must be isolated from feedforward and lateral inputs. In recent human studies [9,10,11,12], we developed a paradigm to measure contextual feedback signals using functional brain imaging and multivariate pattern analysis. We partially masked full-field images with an occluder over the lower-right visual field. The portion of V1 representing the occluder does not receive feedforward inputs that are informative of the scene. Consequently, successful decoding of images in this non-differentially stimulated region is dependent on the content of contextual feedback inputs (and, to a lesser extent, lateral inputs) carrying information about the scene presented to the surrounding visual field. These contextual feedback signals are not explained by eye movements and attentional influences [9,10,11,12]. We have recently shown that contextual feedback signals to V1 generalise across spatial frequencies [11] and can be modelled by line drawings [12]. That is, compared with other visual feature models, behavioural line drawings best describe our mental representation, or internal model, of the missing scene information. Here, we are interested in the spatial precision of this back-projected “line drawing” that higher visual areas communicate to V1.

Our objective was to measure the spatial precision of the contextual feedback signal [9,10,11,12], extending our previous findings [10] in which we found that contextual feedback to superficial layers of V1 has a precision of somewhere between 2 and 6 degrees. There, our primary objective was in identifying the cortical-layer-specific profile of contextual feedback signals, whereas here we parametrically test within this range to more rigorously test the spatial precision of contextual feedback signals. We trained a multivariate pattern classifier to discriminate neuronal population patterns in non-stimulated V1 in response to two partially occluded natural scene images. We then tested the classifier on spatially displaced versions of the same scenes up to 8 degrees. We observed successful generalisability of our brain activity patterns in V1 across ‘shifted’ images. This finding is consistent with the idea that contextual feedback inputs contain global scene features, allowing V1 to process top-down information that spans larger regions of individual natural scenes.

## 2. Materials and Methods

### 2.1. Participants

We paid twenty-eight subjects (19–26 years, 16 females) with normal vision and not currently taking psychoactive medication to participate. We screened subjects for potential contraindications and acquired written informed consent. Our experiment was approved by the local ethics committee of the College of Science and Engineering (University of Glasgow CSE01063). Eight subjects participated in each of experiments one and two, and six subjects in experiments three and four, with the stimuli changing between experiments (see below) and no subject participating in more than one experiment. We excluded one subject from experiments two (*n* = 7) and four (*n* = 5) due to chance-level multivariate pattern classification during feedforward stimulation. Data are reported for the remaining 26 subjects and are pooled across the experiments for the data analysis.

### 2.2. Stimuli

**Figure 1:**
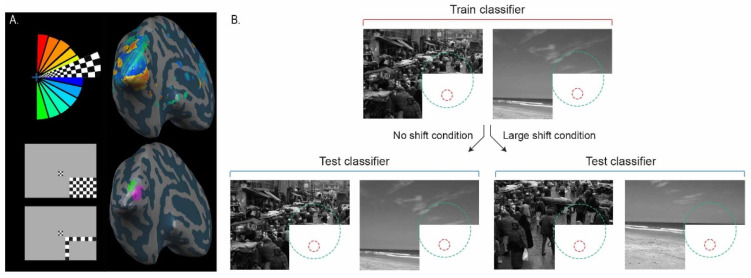
Retinotopic mapping of non-stimulated human V1 shown on the inflated hemisphere of one subject. (**A**) Standard polar wedge retinotopic mapping procedures mapped early visual areas. ‘Target’ and ‘surround’ checkerboard mapping conditions localised the region of V1 responding to the occluded lower-right image quadrant (V1, purple; V2, green). (**B**) Schematic showing the cross-classification procedure. We took voxels only responding to the white mask in the lower-right image quadrant. We entered these data to the classifier, training the classifier on images at one ‘shift’ and testing the classifier at a different ‘shift’. Schematic feedforward and feedback (predictive) receptive fields are shown in red and green, respectively (not to scale), to highlight the hypothesised interactions between non-stimulated neurons and parametrically stimulated neurons feeding back to the occluded region.

In each of four experiments, we presented two images at three different ‘spatial shifts’ to make six conditions. We used different natural scene images in each of the experiments in order to generalise across stimuli. We used the following ‘spatial shifts’: 0 degrees (original image), 2 and 8 degrees (experiments one and two), and 3 and 7 degrees (experiments three and four). Using cross-classification analyses (see below), these stimuli allowed us to test 2, 3, 4, 6, 7, and 8 degrees of spatial shift. A spatial shift refers to an image being spatially displaced leftwards and upwards to create a new version of the original image. This has the effect of introducing image information beyond the boundary of the original image at the right and lower image borders and cropping information present in the original image at the left and upper image borders. Images were greyscale and matched for the Fourier amplitude (experiments 2, 3, and 4) using SHINE toolbox for MATLAB [13]. Images spanned 30° × 20° of the visual angle, which was the full size of the presentation field. There was a white occluder rectangle spanning 13° × 8° presented over the lower-right image quadrant during all image trials. We used three contrast-reversing checkerboards (4 Hz) to map the cortical representation of the occluded image portion, henceforth ‘target’, ‘surround 1′, and ‘surround 2′ (Figure 1). We designed the mapping stimuli to localise in V1 a non-stimulated region, receiving only uninformative feedforward inputs. Brain activity patterns read out from this region contain signals related to contextual feedback and, to a lesser extent, lateral inputs. We minimise the contribution of lateral inputs by excluding voxels responding to our ‘surround’ checkerboard conditions. See [9,10,11,12] for previous use of this paradigm. We refer to non-stimulated ‘feedback’ and stimulated ‘feedforward+’ conditions for clarity, although the feedforward condition also contains feedback signals. We use the ‘+’ to indicate that feedforward will automatically trigger feedback processing. Feedback signals may include lateral signals from within V1 and cortical feedback from any other cortical area, but, importantly, the classical receptive field does not receive informative direct thalamic input.

### 2.3. Design and Procedure

During a block design fMRI experiment, we generated stimuli using Presentation software (version 10.3., Neurobehavioral Systems, Inc., Berkeley, CA, USA) and presented visual stimulation using an MR-compatible binocular goggle system (NordicNeuroLab (NNL), Bergen, Norway; [14]). We monitored the eye movements of the right eye using an NNL Eye Tracking Camera and ViewPoint EyeTracker^®^ by Arrington Research (Scottsdale, AZ, USA) to ensure that subjects maintained fixation on the central checkerboard. Experimental runs consisted of six image conditions and three occluder mapping conditions in each run. Conditions were three ‘shifted’ versions of two natural scene images or ‘target’ and two ‘surround’ mapping conditions (described above) or a fixation baseline.

In each 12 s trial, we flashed the stimulus image on and off (200 ms on/200 ms off) 30 times in order to increase the signal-to-noise ratio [11]. We randomly ordered image trials within each sequence of 6 images (lasted 72 s; 6 × 12 s). We presented a 12 s fixation period before and after each sequence of trials for a baseline. Each of the four experimental runs lasted 12 min 48 s, consisting of six image sequences and the mapping sequence (with Target and the two Surround checkerboards). To maintain attention and reduce eye movements, subjects fixated on a central checkerboard and reported a fixation colour change with a button press.

### 2.4. Retinotopic Mapping of Early Visual Areas

We mapped V1–V3 using a standard phase-encoded polar angle protocol using identical standard parameters to the main functional runs [15,16]. A wedge-shaped checkerboard rotated clockwise around the central fixation point, starting at the right horizontal meridian.

### 2.5. MRI Data Acquisition

We acquired the data at the Centre for Cognitive Neuroimaging, Glasgow, on a 3T Siemens Tim Trio MRI scanner (Siemens, Erlangen, Germany) using a 12-channel head coil. We measured blood oxygen level dependent (BOLD) signals using an echo planar imaging (EPI) sequence for parallel imaging using the following parameters: 18 slices (oriented on the AC–PC plane to cover the early visual cortex); repetition time (TR), 1 s; echo time (TE), 30 ms; flip angle (FA), 62°; field of view (FOV), 210 mm; isotropic voxel size 3 mm; gap thickness, 10% (0.3 mm), PACE motion correction; iPAT factor 2. We also acquired high-resolution T1-weighted anatomical scans: TR, 2 s; TE, 4.38 ms; FA 15°; FOV, 240 mm; isotropic voxel size, 1 mm; 192 volumes.

### 2.6. Data Analysis

We performed fMRI data analysis using BrainVoyager software 2.8 (Brain Innovation, Maastricht, The Netherlands) and MATLAB 2012a (The MathWorks Inc., Natick, MA, USA). We discarded the first two volumes of each functional run to avoid T1 saturation effects. We performed a standard pre-processing protocol: slice scan time correction using sync interpolation, standard three-dimensional motion correction to adjust for head movements as well as linear-trend removal and temporal high-pass filtering at 0.006 Hz. After alignment with the anatomical scan, we transformed individual datasets into Talairach space [17] and overlaid them on inflated cortical hemispheres.

### 2.7. Retinotopic Mapping

We used a cross-correlation analysis for the retinotopic-mapping experiment. We used the predicted hemodynamic signal time course for the first 1/8th of a stimulation cycle (32 volumes/4 volumes per predictor) and slowly shifted this reference function clockwise in time (4 volumes corresponding to 45° visual angle, [16]). Activation maps indicate the highest correlation in cross correlation with the lag specific colour code indicating the visual polar angle that it best represents (Figure 1A).

### 2.8. Cortical Surface Reconstruction and Region-of-Interest (ROI) Definition

We reconstructed both cortical hemispheres for all subjects using high-resolution anatomical scans [18]. We performed an inhomogeneity correction of signal intensity before automatically segmenting the white and grey matter border. Then, we identified the borders between early visual areas (using retinotopic mapping data) before localising the non-stimulated region of V1 by projecting the responses to the checkerboard stimuli using a generalised linear model (GLM) approach. This allowed us to select voxels responding to the occluded image quadrant and test how they are modulated by cortical feedback signals responding to the surrounding image context. We contrasted the response to the Target checkerboard against the Near Surround checkerboard (as in [9,10,11,12]). This process minimised spillover contributions from voxels stimulated by the image in a feedforward manner that could be transmitted into our non-stimulated ROI by lateral processing. To localise the region of interest in the stimulated right hemisphere, we used a GLM approach to identify voxels responding to all six image conditions.

### 2.9. Multivariate Pattern Classification Analysis

On a per subject and per run basis, we applied a GLM to estimate single trial response amplitudes (beta values) for each voxel time course. The design matrix relating to single-trial response estimation consisted of one regressor per trial all together for one run. We entered voxel responses into a support vector machine (SVM) linear classifier using LIBSVM MATLAB toolbox [19]. We trained the classifier to discriminate between two scenes in each spatial shift condition (e.g., image 1 versus image 2 at 4 degrees, at 6 degrees, and so forth) on three functional runs and tested it on the residual fourth run (leave-one-run-out cross-validation). The classifier used single-trial responses for training and was tested on single-trial or average (across the block) activity patterns for each of the 6 trial types. During a cross-classification procedure, we trained the classifier on images at one ‘shift’ and tested the classifier at a different ‘shift’. In order to attain a robust average value and to examine classifier performance with shuffled labels, we used bootstrapping and permutation as we have previously carried out [11]. Per subject, we bootstrapped classifier performances with 1000 samples, which provided an estimate of individual subject means. Then, we bootstrapped these individual means in order to estimate the group means and confidence intervals (CIs) for each condition (1000 samples). Our confidence intervals covered the middle 95% of the distribution, and we considered classifier performances to be significantly above a chance of 50% (due to classifying two conditions) only if the confidence intervals did not contain chance-level performance. We tested differences between group performances using a permutation test (1000 samples) of the differences between group means. This consisted of shuffling the observed values across conditions 1000 times and computing absolute differences between conditions. We classified groups to be significantly different if the actual observed difference was in the top 5% of the differences distribution [11].

## 3. Results

### 3.1. Contextual Feedback Processing and Comparison with Feedforward Classification

**Figure 2:**
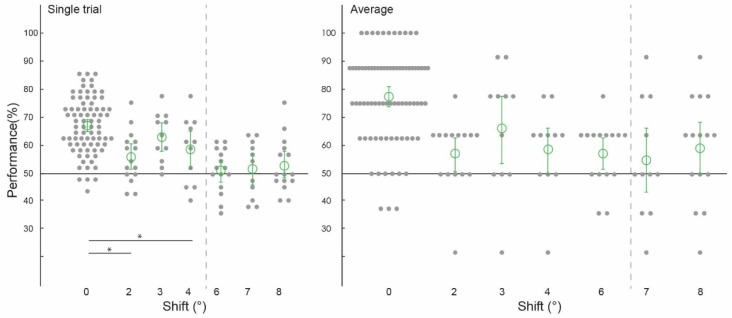
Spatial precision of contextual feedback signals. Pattern classifier performance (percent correct) across experiments and subjects for single-trial and average analyses. The classifier was trained and tested on voxel patterns from the non-stimulated region of V1, responding to the lower-right image quadrant processing the white occluder. (All conditions to the left of the grey dashed line were statistically significant, significant differences between conditions marked with an asterisk).

**Figure 3:**
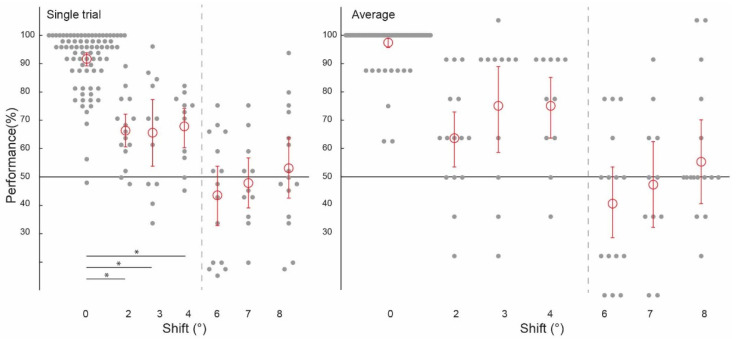
Spatial precision of information patterns in feedforward+ stimulated V1. Pattern classifier performance (percent correct) across experiments and subjects for single-trial and average analyses. The classifier was trained and tested on voxel patterns from a stimulated region of V1 viewing the lower-left image location. (All conditions to the left of the grey dashed line were statistically significant, significant differences between conditions marked with an asterisk).

**Figure 4:**
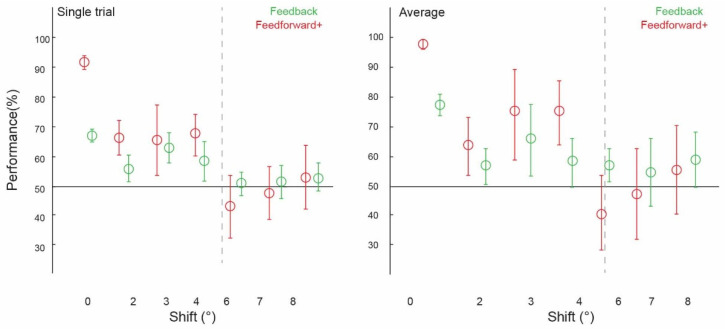
Spatial precision of information patterns in V1, averaged within feedback and feedforward+ conditions (shown in Figure 2 and Figure 3). Pattern classifier performance (percent correct) across experiments and subjects for single-trial and average analyses.

Prior to testing the spatial precision of contextual feedback, we tested for a confirmation of our previous findings. That is, can we discriminate the surrounding image from a non-stimulated region of V1? Across subjects, image classification was significantly above chance in both single-trial classification (ST) (67.12%, confidence interval (CI) [0.0224, 0.0216]) and average block classification (AB) (77.40%, (CI) [0.0369, 0.0353]; Figure 2, ‘0 degrees shift’). (We call this condition ‘0 degrees shift’ because the classifier was trained and tested on the same images). This is a conceptual replication of our previous findings where we also found contextual feedback of natural scene features to non-stimulated V1 [9,10,11,12], however here using different image stimuli. For comparison with contextual feedback processing in non-stimulated V1, we also tested how the classifier discriminates images in the feedforward+ (stimulated) condition. Here, we expect image-specific neuronal information patterns in V1 that the classifier can reliably label as different; the classifier was able to discriminate two images, performing at [ST]: 91.69%, (CI) [0.0251, 0.0211]; and (AB): 97.44%, (CI) [0.0176, 0.0144] (Figure 3 and Figure 4, ‘0 degrees shift’).

### 3.2. Spatial Precision of Cortical Feedback Processing

We collapsed the data across experiments one and two, which used different subjects and stimuli but the same spatial shifts (2 degrees, 6 degrees, and 8 degrees), and across experiments three and four (both using 3 degrees, 4degrees, and 7 degrees conditions). We were able to train our classifier to discriminate two images and test it on shifted versions of the same two images up to 4 degrees, but not at 6 degrees, 7 degrees, or 8 degrees (Figure 2). Classifier performance was higher in the 0 degrees shift condition than in the 2 degrees (*p* = 0.002) and 4 degrees shift (*p* = 0.01) conditions, with the permuted *p* value at 0.06 when comparing the 0 degrees and 3 degrees shift conditions. Specifically, image classification was significantly above chance for the ‘2 degrees shift’ (both single-trial classification (ST): 55.56%, confidence interval (CI) [0.0403, 0.0431]; and average block classification (AB): 56.67%, (CI) [0.0583, 0.0500]), ‘3 degrees shift’ (both single-trial classification (ST): 61.93%, (CI) [0.0436, 0.0455]; and average block classification (AB): 64.77%, (CI) 0.1136, 0.1023), and ‘4 degrees shift’ ((ST): 57.95%, (CI) [0.0606, 0.0587]; (AB): 57.95%, CI [0.0909, 0.0682]), but not for the ‘6 degrees shift’ ((ST): 51.25%, (CI) [0.0347, 0.0347]; (AB): 56.67%, (CI) [0.0500, 0.0500]), ‘7 degrees shift’ ((ST): 51.70%, (CI) [0.0511, 0.0492]; (AB): 54.55%, CI [0.1023, 0.1136]), or ‘8 degrees shift’ ((ST): 52.64%, (CI) [0.0389, 0.0458]; (AB): 58.33%, CI [0.0833, 0.0833]).

### 3.3. Spatial Precision of Feedforward + Processing

Using voxels from the feedforward stimulated right hemisphere, and again collapsing across data in experiments one and two using the same values of ‘spatial shift’ (2 degrees, 6 degrees and 8 degrees), and experiments three and four (3 degrees, 4 degrees and 7 degrees), we observed a similar pattern of classifier performances as in the feedback conditions. We were again able to train our classifier to discriminate two images and test it on shifted versions of the same two images up to 4 degrees, but not at 6 degrees, 7 degrees, or 8 degrees (Figure 3). As expected, because the classifier was trained and tested on the same images, classifier performance was significantly higher in the 0 degrees shift condition than in the 2 degrees, 3 degrees, and 4 degrees shift conditions (all *p* < 0.001). Classification data is as follows: performance was significantly above chance for the ‘2 degrees shift’ ((ST): 65.00%, confidence interval (CI) [0.0514, 0.0528]; (AB): 62.50%, (CI) [0.0917, 0.0917]), ‘3 degrees shift’ ((ST): 64.39%, (CI) [0.1098, 0.1042]; (AB): 72.73%, (CI) 0.1477, 0.1250), and ‘4 degrees shift’ ((ST): 66.29%, (CI) [0.0663, 0.0606]; (AB): 72.73%, CI [0.1023, 0.0909]), but not for the ‘6 degrees shift’ ((ST): 44.31%, (CI) [0.0931, 0.0917]; (AB): 41.67%, (CI) [0.1167, 0.1167]), ‘7 degrees shift’ ((ST): 48.30%, (CI) [0.0795, 0.0814]; (AB): 47.73%, CI [0.1364, 0.1250]), or ‘8 degrees shift’ ((ST): 52.78%, (CI) [0.0944, 0.0986]; (AB): 55.00%, CI [0.1333, 0.1333]).

## 4. Discussion

Using fMRI, we tested the spatial precision of contextual feedback inputs to V1 during scene processing. We measured spatial precision by generalising brain activity patterns from a region of non-stimulated V1 across parametrically spatially displaced stimulus images up to 8 degrees. fMRI activity patterns in cortical feedback signals predicted scene-specific features in V1 with a precision of approximately 4 degrees. This result is in line with anatomical evidence from the non-human primate visual cortex that higher areas with larger receptive field sizes, for example, area V4, send feedback to V1 [20,21] and reveals using functional brain imaging that these contextual inputs carry more global information about the scene compared with more locally tuned feedforward inputs. PRF measures estimate the receptive field size of about 2 degrees in area V2 and 4 degrees in area V4. Our results are consistent with stimuli comprising of static visual scenes and objects receiving meaningful feedback information from areas up to V4 (V2, V3, V4; [22]). Furthermore, we have replicated our previous findings of contextual feedback to non-stimulated V1 [9,10,11,12] and tested additional parameters of precision than in [10], where we could generalise across 2 degrees but not 6 degrees, suggesting that the spatial precision of cortical feedback signals is within this range.

By calculating the spatial resolution of the contextual feedback signal to V1 during scene processing and comparing this value with receptive field properties along the visual processing hierarchy, we can speculate about the areas from which our feedback signals arise. The structural organisation of the primate visual system comprises a richly interconnected hierarchy of areas with feedforward and feedback pathways bi-directionally communicating information, with dense intra-area connections at all levels [23]. Given that our contextual feedback signal had a precision of up to approximately 4 degrees, we can estimate that with our paradigm and orthogonal task, we captured feedback originating in early and mid-level extrastriate visual areas that have classical feedforward receptive fields of this size. One interpretation of our findings is that we measured cortical feedback signals from higher areas processing larger regions of the visual field than V1 that feed back to V1 the output of their perceptual computations, which could include object segmentation, border ownership, and spatial integration in natural scenes [24]. Given that our stimuli were occluded, our results are also in line with the visual system’s ability to infer and interpret the missing content of sensory inputs using predictive mechanisms, e.g., [25]. We recently showed that contextual feedback signals to V1 can be modelled by line drawings [12]. Compared with other visual feature models, behavioural line drawings best described internal models of the occluded scene information, but it was not known how spatially precisely these missing scene features were filled in. Precise line continuation might speak towards lateral communication within V1 in general (though we controlled for this), but this communication would still be bound by V1’s spatial resolution. Since our finding of 4-degree precision is more compatible with the receptive field of higher areas, we can exclude that this signal is purely line continuation from within V1 (i.e., it probably contains higher-level information). This higher-level information could, for example, describe which side of a borderline belongs to the foreground object or the background. Such contextual information would be valuable even at a precision of 4 degrees. Furthermore, the presence of feedforward information does not lead to higher classification when any shift is added, which additionally suggests that V1 processes information at a high resolution and by itself might not do any inference when the information is not physically consistent (e.g., when a line is broken).

Spatially shifting the stimulus image is perceptually similar to different viewpoints of the same scene, for example, during eye movements. During eye movements, global image features might remain largely unchanged, but the precise retinotopic projection onto V1 differs with each saccade. Here, the expected local features behind the occluder change with each image shift, but the overall scene content remains the same. In line with this, our results might relate to a form of gist processing where local variability in V1 is cancelled by lower-resolution feedback from higher areas, possibly with high-level feedback signals more broadly spanning the visual field and affecting how the entire scene is processed in V1. In our feedforward+ condition, we measured the spatial precision of neuronal response patterns to image features (as opposed to masked image features in the feedback condition). Here, the data we entered to our classification analyses were taken from the stimulated right hemisphere, responding to the lower-left visual field. We found that we were again able to train our classifier to discriminate two images and test it on shifted versions of the same two images up to 4 degrees, but not beyond. One might expect not to see generalisation across stimuli in the feedforward+ condition because of the small, locally tuned response profiles of V1 neurons. This local tuning could, in theory, mean that even small shifts in the image would change the inputs, and neuronal patterns would no longer carry enough shared information to withstand training and testing on different images during cross-classification. We did find the expected effect of near-ceiling classification in the 0 degree (i.e., non-shifted) feedforward+ condition, in line with high-precision activation patterns in feedforward stimulation. However, this region receives contextual feedback signals as well as being feedforward-driven; hence, we can attribute the ability to be able to generalise across feedforward conditions to the presence of feedback information in this condition. Finding consistent feedback-related shift-invariant decoding in V1 speaks for a general computational function during normal vision. High-resolution processing information might be contextualised with low-precision feedback up to 4 degrees of visual angle. In previous findings, we found this contextual feedback to target the superficial layers of V1 [10], consistent with the evidence that feedback signals target the apical dendrites in cortical layer 1 of pyramidal cells in deeper layers [26,27].

## 5. Conclusions

We conclude that cortical feedback signals in V1 can generalise to 4 degrees in visual angle. The feedback generalisation is accessible during bottom-up visual stimulation when automatic cortical top-down processing is coexisting, and during top-down processing when bottom-up stimulation is masked. Our data can contribute to constraining future animal and modelling studies investigating how feedforward and feedback signals are combined in perception, e.g., [28,29]. Neuronal data and models of dendritic computation and biologically informed machine learning will advance our understanding of the hierarchical exchange of sensory and internally driven data mapped onto the laminar architecture of the cortex.

## Data Availability

Data and code are available upon request.
